# Influence of gingival thickness on soft tissue changes after immediate implants and immediate provisionalization in the esthetic zone: an ambispective cohort study

**DOI:** 10.3389/fmed.2026.1888629

**Published:** 2026-08-19

**Authors:** Wang Maoxia, Mo Anchun, Wu Yan

**Affiliations:** 1 West China School of Public Health and West China Fourth Hospital, Sichuan University, Chengdu, China; 2 State Key Laboratory of Oral Diseases, National Center for Stomatology, National Clinical Research Center for Oral Disease, Department of Oral Implantology, West China Hospital of Stomatology, Sichuan University, Chengdu, China; 3 State Key Laboratory of Oral Diseases, National Center for Stomatology, National Clinical Research Center for Oral Diseases, Department of West Outpatient, West China Hospital of Stomatology, Sichuan University, Chengdu, China

**Keywords:** gingival recession, gingival thickness, immediate implants, immediate provisional restoration, soft tissue changes

## Abstract

**Background:**

To evaluate the influence of gingival thickness on soft tissue changes after immediate implant placement and provisionalization (IIPP).

**Methods:**

Twenty-two patients requiring IIPP were categorized into two groups based on the visibility of a periodontal probe in the sulcus: thin phenotype and thick phenotype. Gingival thickness was measured preoperatively, while midfacial recession, papilla recession, and horizontal contour collapse were evaluated at 3 and 6 months postoperatively. Comparative analyses of soft tissue alterations between the groups were conducted, alongside an examination of the correlation between gingival thickness and soft tissue changes.

**Results:**

A total of twenty-two patients, encompassing 30 sites, completed the 6-month follow-up period. At 6 months, the thin phenotype group exhibited a midfacial recession of 0.73 ± 0.69 mm, compared to 0.89 ± 0.52 mm in the thick phenotype group. No statistically significant differences in soft tissue alterations were observed between the groups, and no statistically significant association correlation was identified between gingival thickness and soft tissue changes.

**Conclusion:**

Within the constraints of this study, no statistically significant differences in soft tissue alterations were observed between different gingival phenotype groups at 6 months following IIPP. Since the study was powered to detect a clinically relevant difference of 1 mm, it lacked the sensitivity to definitively confirm or rule out the more subtle difference observed.

## 1 Introduction

Since immediate implant placement and provisionalization (IIPP) was firstly reported by Wöhrle in 1998, it has been widely used in the esthetic zone given the advantages in minimizing the treatment duration and improving patient satisfaction (1–3). Much clinical evidence has corroborated the effectiveness of IIPP (4, 5). Lang et al. reported a 2-year survival rate of 98.4% on 2098 immediate implants in a systematic review, while no difference was found between different types of loading (6). Despite the high survival rate of IIPP, it remains challenging in achieving predictable esthetic outcomes with IIPP. As immediate implant placement is not able to prevent the physiologic bone resorption following tooth extraction (7, 8), immediate implants are associated with a high risk of soft tissue recession (9, 10). A systematic review showed that a recession of the midfacial soft tissue (> 1 mm) was observed in 9–41% of immediate implants during a follow-up of 1–3 years (11). Ac-cording to a 10-year prospective study, 33% of immediate implants displayed advanced midfacial recession (≥1 mm) (12).

Various factors, such as the implant, socket anatomy, surgical protocol, the use of provisional restorations, and gingival thickness, are considered to be related to the soft tissue recession of immediate implants (2, 10, 13–16). Among these factors, the role of gingival thickness remains controversial. Gingival thickness was once believed to be an important factor influencing the esthetic outcomes of IIPP. A 2- to 8-year follow-up prospective study demonstrated that the sites with thin phenotype exhibited more facial gingival recession following IIPP than thick biotype (17). Besides, the consensus statement from the 5th International Team for Implantology (ITI) Consensus Conference pointed out that the site with thin soft tissue was not suitable for immediate implant placement (18). However, some other studies presented conflicting results. Cabello et al. conducted a prospective study to trace the soft tissue alterations following IIPP and found that gingival biotype was not correlated with the soft tissue changes during a 1-year follow-up period (19). Similar results were also reported by a retrospective study of 42 implants with a follow-up ranging from 6 to 50 months (9). Furthermore, a recent systematic review indicated that the thin gingival phenotype did not have a negative impact on soft tissue alterations following IIPP (20). Despite this debate, it is remarkable that the clinical evidence about the soft tissue alteration following IIPP is still insufficient. Since previous studies have predominantly relied on periodontal probes for two-dimensional measurements of soft tissue changes in a single plane, there remains a paucity of research investigating two-dimensional soft tissue alterations across multiple planes following immediate implant placement and provisionalization in patients with varying gingival thickness. As digital technology advances, the digital surface scan could be used to trace the dynamic alterations of soft tissue and was thought to be the most accurate method to evaluate profilometric tissue changes (21). To the best of our knowledge, there was a lack of study assessing the soft tissue dynamic alterations of different gingival phenotypes following IIPP using this digital method.

Hence, the aim of the study was to evaluate the influence of gingival thickness on soft tissue changes following IIPP. The null hypothesis was that there is no difference between different gingival phenotypes. This study contributes to a more comprehensive two-dimensional assessment of soft tissue changes following immediate implant placement and provisionalization across varying gingival thicknesses.

## 2 Materials and methods

The STROBE guidelines for reporting observational cohort studies were followed in the present study.

### 2.1 Study design

This study was designed as an ambispective cohort study and a duration period of 6 months. The study was approved by the ethical committee of West China Hospital of Stomatology, Sichuan University (WCH-SIRB-D-2017-113). The study was performed at the Department of implant dentistry, West China Hospital of Stomatology, Sichuan University, China. The study adhered to the Declaration of Helsinki guidelines. Participants were included from September 2016 to December 2020. The retrospective study period was from September 2016 to December 2017, and the prospective study period was from January 2018 to December 2020. Informed consent was obtained from all patients.

### 2.2 Eligibility criteria

#### 2.2.1 Inclusion criteria

- Male or female patients at least 18 years of age.
- Implant site in the anterior maxilla.
- Intact facial bone wall after tooth extraction and a sufficient bone volume(a buccolingual ridge width of > 6 mm).
- Adequate bone volume apically of the root to ensure good primary stability (a minimum of 3–5 mm of bone height in the apical region).
- A minimum keratinized tissue width of 2 mm.
- Good general health.
- Good oral hygiene (full-mouth plaque score, FMPS < 25%; full-mouth bleeding score, FMBS of <25%).

#### 2.2.2 Eligibility criteria

- Incomplete facial bone wall after tooth extraction.
- The insertion torque < 35 Ncm.
- Uncontrolled systematic diseases.
- Presence of acute infection in the surgical site.
- Smoking.
- Known allergic to the materials used in the product.
- Women in pregnancy or lactation.
- Bruxism.

### 2.3 Clinical procedures

All patients were treated following the same clinical protocol. Before surgery, a cone-beam computed tomography (CBCT) scan (3DAccuitomo 170, J. Morita Mfg. Corp., Kyoto, Japan) and an intraoral scan (3Shape TRIOS®, 3Shape, Copenhagen K, Denmark) were con-ducted. The CBCT used these settings: 90 kV, 5 mA, 17.5 s acquisition time, 140 mm FOV diameter, 100 mm FOV height, and 0.25 mm voxel size. The standard tessellation language (STL) file of the intraoral scan was superimposed on the preoperative CBCT images in a dental software (3Shape Dental System, 3Shape, Copenhagen K, Denmark), allowing for digital implant design in an ideal position (11, 22), and a surgical template was then 3D printed (ProJet MJP 2500Plus, 3D Systems, Inc., Rock Hill, USA).

**Figure 1:**
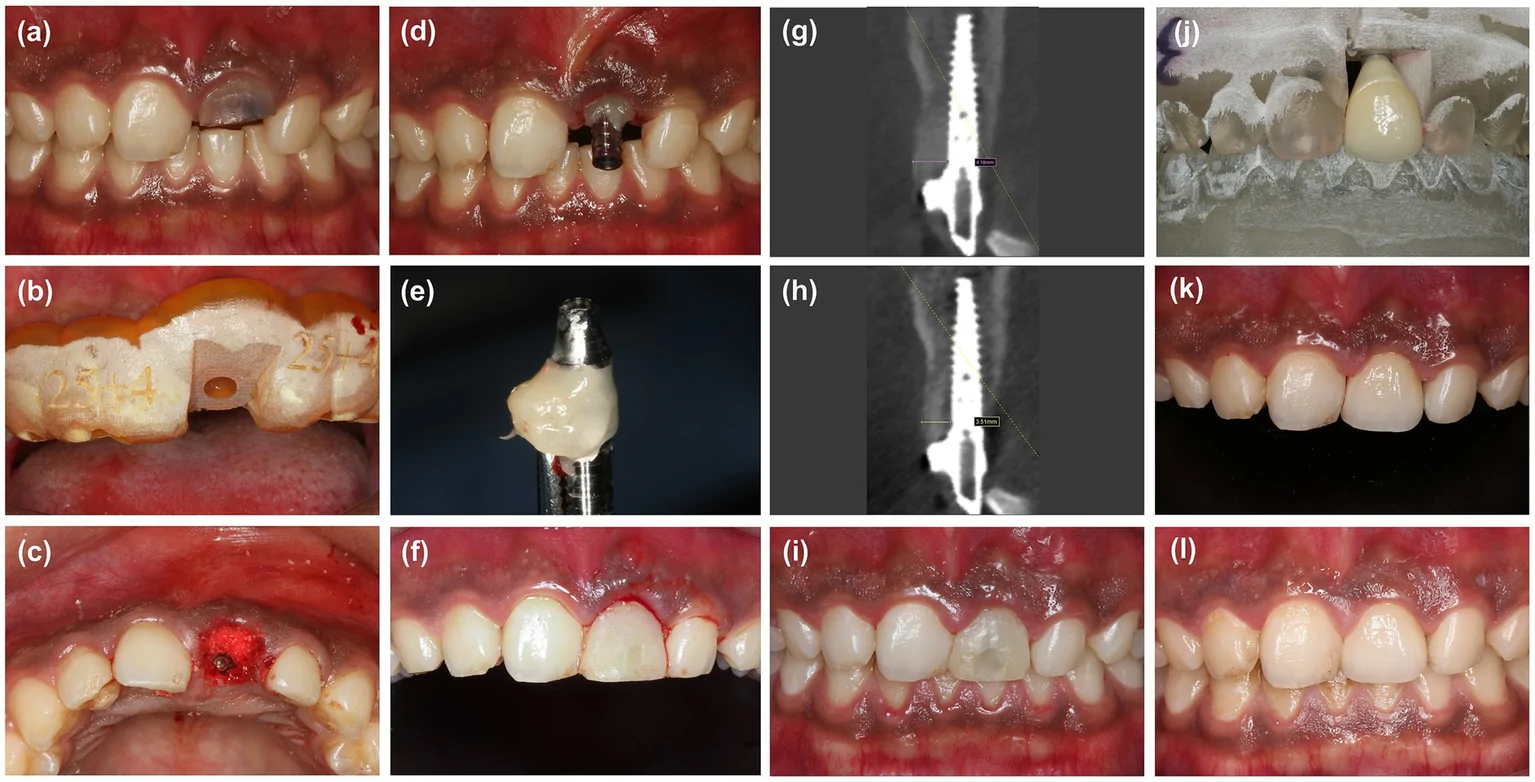
**(a)** Intraoral view before the surgery. **(b,c)** Implant placement guided by a surgical template. **(d–f)** Immediate provisionalization. **(g)** CBCT image immediate post-operation. **(h)** CBCT image at 3 months. **(i)** Intraoral view at 3 months. **(j,k)** Delivery of the final prosthesis. **(l)** Intraoral view at 6 months.

The surgery procedure was performed by an experienced surgeon (AM). Prophylactic oral antibiotics (2 g amoxicillin) were given to all patients one hour before surgery. Minimally invasive tooth extraction was performed after local anesthesia. Guided by the surgical templates, the implants of proper size (NobelActvie, Nobel Biocare, Göteborg, Sweden) were placed in the socket following a flapless protocol. The jumping gap between the buccal bone wall and implant surface was filled with particulate deproteinized bovine bone mineral (DBBM) (Bio-Oss, Geistlich Pharma AG, Wolhusen, Switzerland). When the insertion torque of at least 35 Ncm was obtained, implant im-pression was performed to fabricate screw-retained provisional restorations, which would be connected to the implant within 24 h (Figure 1). The tightening torque of the provisional restorations was 15 Ncm, and occlusal adjustment was performed to avoid any functional loading (23). Anti-biotics (amoxicillin 500 mg three times a day) were used prophylactically postoperatively until the third day postoperatively, and 0.12% chlorhexidine mouth rinses (twice a day) were prescribed for 2 weeks.

**Figure 2:**
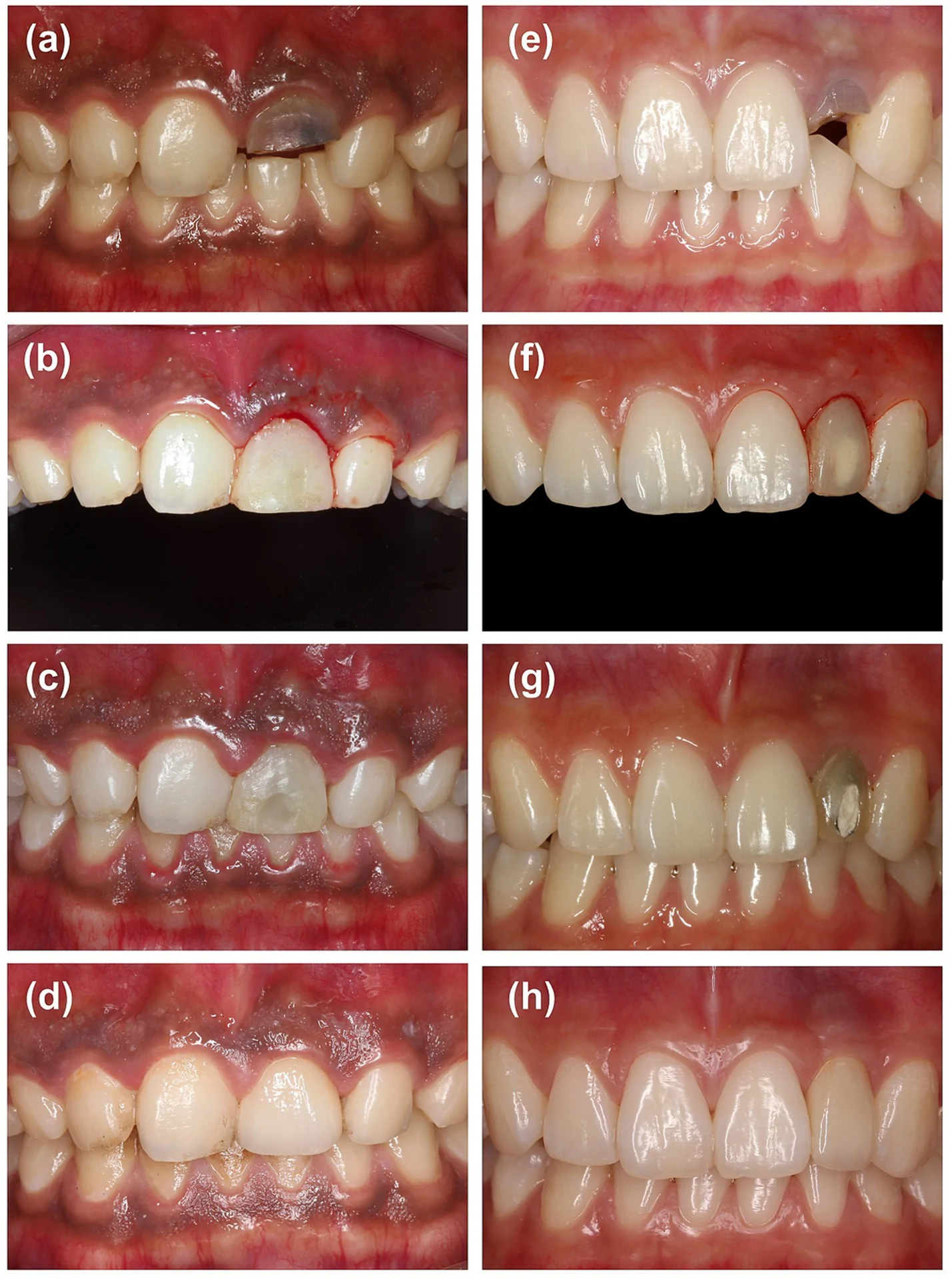
**(a)** Intraoral view before the surgery (thick phenotype). **(b)** Immediate provisionalization (thick phenotype). **(c)** Intraoral view of provisional prosthesis at 3 months (thick phenotype). **(d)** Intraoral view of final prosthesis at 6 months (thick phenotype). **(e)** Intraoral view before the surgery (thin phenotype). **(f)** Immediate provisionalization (thin phenotype). **(g)** Intraoral view of provisional prosthesis at 3 months (thin phenotype). **(h)** Intraoral view of final prosthesis at 6 months (thin phenotype).

At 3 months, patients were recalled for the final impression. The final restorations were manufactured replicating the emergence profile of the provisional restorations (24). At 4 months, the final restorations were delivered. All restorative procedures were performed by the same blinded prosthodontist. Patients were re-visited at 1 week, 3 months and 6 months to record the healing process (Figure 2).

### 2.4 Assessment

#### 2.4.1 Clinical assessment

Patients were revisited at 1 week, 3 months, and 6 months. Implant survival rate and complications (such as infection and loosening of provisional restorations) were recorded.

#### 2.4.2 Analysis of soft tissue alterations

**Figure 3:**
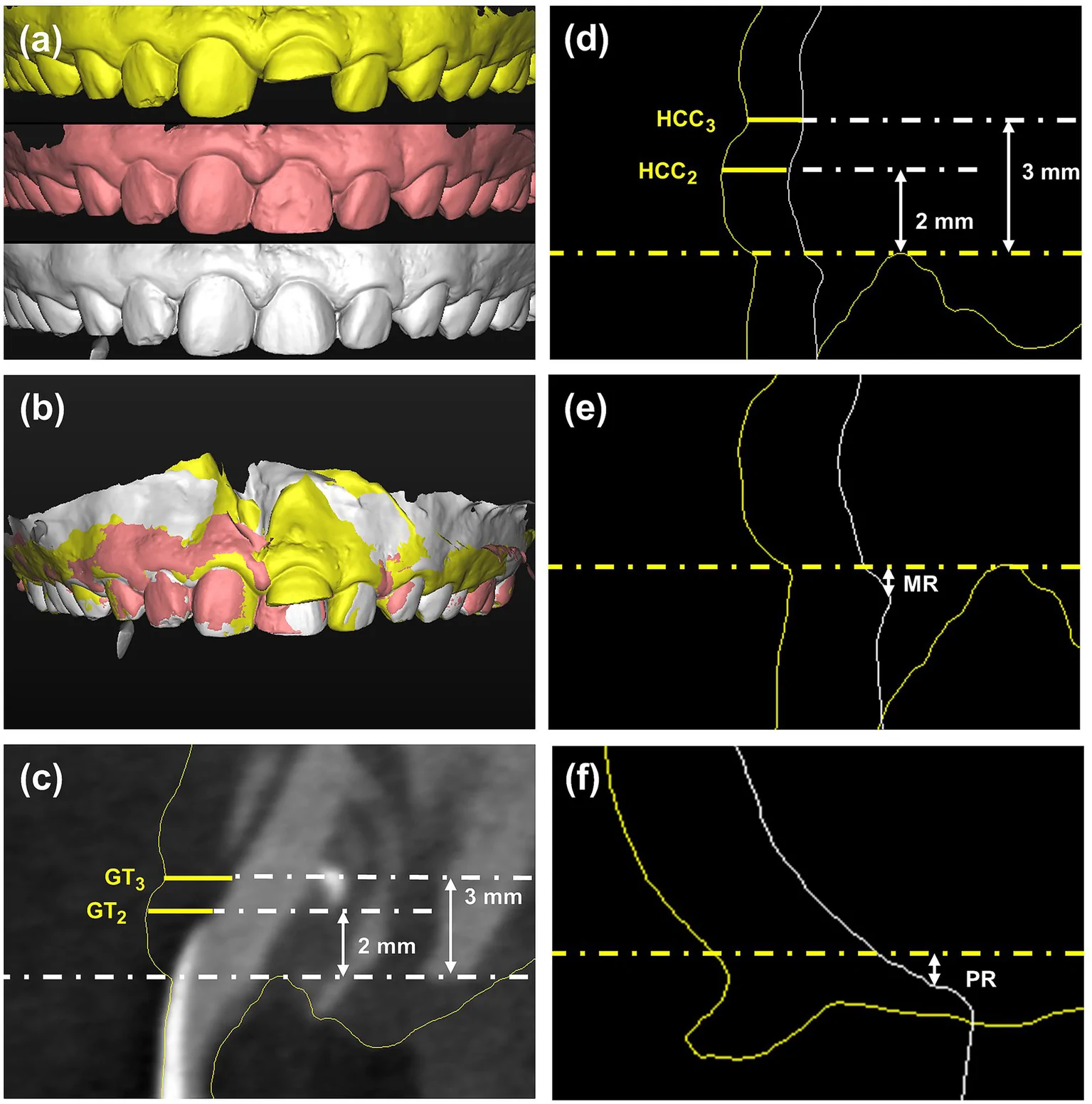
**(a,b)** STL files of the intraoral scan at T_1_ (red) and T_2_ (white) were superimposed on the intraoral scan at T_0_ (yellow). **(c)** Gingival thickness (GT) was measured as the horizontal distance between the contour of T_0_ (yellow) and the tooth surface of the preoperative CBCT images measured at two levels (2 and 3 mm apical to the gingival zenith at T_0_: GT2 and GT3). **(d)** Horizontal contour collapse (HCC) was measured as the horizontal distance between the contour of T_0_ and T_1_/T_2_ at two levels (2 and 3 mm apical to the gingival zenith at T0: HCC_2_ and HCC_3_). **(e)** The midfacial recession (MR) was measured as the vertical distance between the gingival zenith at T_0_ and T_1_/T_2_. **(f)** The papilla recession (PR) was measured as the vertical distance between the top of the papilla at T_0_ and T_1_/T_2_. According to the location of the papilla, the PR was labeled as mesial papilla recession (MPR) and distal papillary recession (DPR).

Intraoral scans were performed before surgery (T_0_), 3 months (T_1_), and 6 months post-surgery (T_2_) by the same blinded prosthodontist. The STL file of intraoral scan at T_0_ was superimposed on the preoperative CBCT images in Simplant software (Simplant Pro 17.01, Dentsply Sirona, Pennsylvania, USA). The STL files of the intraoral scan at T_1_ and T_2_ were then superimposed on the intraoral scan at T_0_, while the same anatomical structures of adjacent teeth were selected as registration points (Figure 3).

The following parameters were measured:

- Midfacial recession (MR): The bucco-oral cross-sectional image at the midfacial gingival margin of the failing tooth was selected for measurements, while the long axis of the tooth was set as the Y-axis. The MR at 3 months/6 months was measured as the vertical distance between the gingival zenith at T_0_ and T_1_/T_2_, respectively.
- Horizontal contour collapse (HCC): The horizontal distance between the contour of T_0_ and T_1_/T_2_ was measured at two levels (2 and 3 mm apical to the gingival zenith at T0: HCC_2_ and HCC_3_).
- Gingival thickness (GT): The horizontal distance between the contour of T_0_ and the tooth surface of preoperative CBCT images measured at two levels (2 and 3 mm apical to the gingival zenith at T_0_: GT_2_ and GT_3_). The MR, HCC, and GT were measured using the same coordinate system.
- Papilla recession (PR): The bucco-oral cross-sectional image at the papilla was selected for measurements. In this cross-section image, the line parallel to the long axis of the tooth was set as the Y-axis. The PR at 3 months/6 months was measured as the vertical distance between the top of the papilla at T_0_ and T_1_/T_2_, respectively. According to the location of the papilla, the PR was labeled as mesial papilla recession (MPR) and distal papillary recession (DPR).

#### 2.4.3 Analysis of bone thickness alterations

Labial bone thickness (LBT): On bucco-oral cross-sectional images perpendicular to the implant, the labial bone thickness at the implant shoulder was measured immediately postoperatively and at 3 months. LBT was defined as the distance between the implant and the labial hard tissue outline.

An experienced and blinded examiner performed all measurements. All measurements were repeated twice and then were averaged. Prior to the data collection, a calibration exercise was conducted to ensure measurement consistency. The examiner performed repeated measurements on an independent set of 10 sites, which were randomly selected and excluded from the final analysis. These measurements were repeated after a 2-week interval to minimize recall bias. All the ICC values exceeded 0.95, indicating a high level of agreement in the measurements.

### 2.5 Outcome variables

- Primary outcome: MR.
- Secondary outcomes: HCC_2_, HCC_3_, MPR, DPR and the correlation between GT and MR, HCC, and PR at 3 or 6 months.

### 2.6 Groups

A periodontal probe was gently probed into the sulcus to evaluate the gingival phenotype of the failing teeth before surgery. Based on the visibility of the periodontal probe, the patients were divided into two groups: thin phenotype (visible) and thick phenotype (invisible) (25).

### 2.7 Sample size calculation

In this study, midfacial recession (MR) was considered as the primary outcome. The objective of the analysis was to test whether there were significant differences in MR between the two groups. Thus, the following hypotheses were adopted: H0, there was no difference in MR between the groups; H1, there was a difference in MR between the groups.

The following variables were used for sample size calculation:

- Clinically relevant difference (Effect size *δ*): set at 1 mm.
- Assessing standard deviation: According to the study of Kan et al., the standard deviation of MR was estimated to be 0.55 mm (17).
- Type I error: *α* = 0.05.
- Type II error: *β* = 0.20.
- Group ratio: 1:1.
- Drop-out rate: 20%.

Sample size calculation was performed using PASS 15 software (PASS 15, NCSS, LLC. Kaysville, Utah, USA). Accounting for a 20% drop-out rate, 16 patients (8 per group) are needed to achieve 80% power to reject the null hypothesis of equal means at a 0.05 significance level with a two-sided two-sample *t*-test.

### 2.8 Statistical analysis

Statistical analyses were conducted using SPSS 20.0 (IBM Company, Armonk, New York). Descriptive statistics summarized the data, with quantitative data presented as means ± SD. The Shapiro–Wilk test checked for normality. Due to some patients contributing multiple sites, Due to some patients contributing multiple sites, site-level data (MR, HCC, GT, LBT, PR) were analyzed using a generalized linear mixed-effect model (GLMM). Categorical variables, including gender, were compared between the two groups using the chi-square test. Age was compared between the two groups using the Mann–Whitney U test. Spearman correlation was used for an exploratory analysis of the relationship between gingival thickness and soft tissue changes; however, the results did not account for data clustering. The significance level was set at 0.05.

## 3 Results

### 3.1 Patients

Table 1 presents the baseline patient demographics and site characteristics. Twenty-two patients with 30 sites were included in this study. No patients dropped out during the follow-up period. No statistically significant differences existed between the two groups in age, gender, tooth positions, and labial bone thickness. The thick phenotype group showed a greater gingival thickness as compared to the thin phenotype group. The GT_2_ values in the thin phenotype group were all less than 1 mm, whereas those in the thick phenotype group were all greater than 1 mm. No complication occurred during the healing period, and the implant survival rate was 100% in both groups.

**Table 1:** Descriptive statistics for each group.

Variable	Thin	Thick	*p* values
Patient demographics			
Male/Female	3/6	6/7	0.674
Mean age ± SD	39.78 ± 15.30	39.62 ± 15.66	0.794
Information about the implant sites			
Location Incisor/Canine	11/3	16/0	0.090
GT_2_	0.87 ± 0.14	1.74 ± 0.43	0.000*
GT_3_	0.89 ± 0.31	1.91 ± 0.48	0.000*
LBT immediately post-operation	2.53 ± 0.67	2.57 ± 0.81	0.711
LBT 3 months post-operation	1.53 ± 0.38	1.59 ± 0.81	0.965

SD, standard deviation; GT_2_, the gingival thickness measured at 2 mm apical to the gingival zenith at baseline; GT_3_, the gingival thickness measured at 3 mm apical to the gingival zenith at baseline. LBT, labial bone thickness.

*Statistically significant.

### 3.2 Primary outcome

The results of MR at 3 months and 6 months were shown in Table 2. At 3 months, the thin phenotype group showed an MR of 0.61 ± 0.58 mm (95% CI: 0.28 to 0.95 mm), while the MR in the thick phenotype group was 0.86 ± 0.71 mm (95% CI: 0.48 to 1.43 mm). At 6 months, the MR was 0.73 ± 0.69 mm (95% CI: 0.33 to 1.13 mm) in the thin phenotype group and 0.89 ± 0.52 mm (95% CI: 0.61 to1.17 mm) in the thick phenotype group. At each time point, no statistically significant difference was observed between the groups (*p* > 0.05).

**Table 2:** Results of soft tissue alterations for each group (mean ± SD, mm).

Parameter	3 months (T_1_)			6 months (T_2_)		
	Thin	Thick	*p* values	Thin	Thick	*p* values
MR	0.61 ± 0.58	0.86 ± 0.71	0.307	0.73 ± 0.69	0.89 ± 0.52	0.485
HCC_2_	1.18 ± 0.55	1.04 ± 0.59	0.829	1.17 ± 0.58	1.05 ± 0.64	0.959
HCC_3_	1.08 ± 0.50	0.88 ± 0.47	0.486	1.08 ± 0.52	0.96 ± 0.54	0.882
MPR	1.00 ± 0.40	0.96 ± 0.49	0.753	1.13 ± 0.57	0.93 ± 0.54	0.779
DPR	0.76 ± 0.39	0.81 ± 0.48	0.638	0.97 ± 0.34	0.78 ± 0.40	0.375

SD, standard deviation; MR, midfacial recession; HCC_2_, the horizontal contour collapse measured at 2 mm apical to the gingival zenith at T_0_; HCC_3_, the horizontal contour collapse measured at 3 mm apical to the gingival zenith at T_0_; MPR, mesial papilla recession; DPR, distal papillary recession.

### 3.3 Secondary outcomes

The results of soft tissue alterations for each group were displayed in Table 2. There was no statistically significant difference in HCC_2_, HCC_3_, MPR, and DPR between the groups (*p* > 0.05). The correlation between GT and soft tissue alterations (MR, HCC_2_, HCC_3_, MPR, and DPR) was shown in Table 3. There was no significant correlation existed (*p* > 0.05). The overall soft tissue alterations at 3 months and 6 months were presented in Supplementary Table S1. At 3 months and 6 months, the values of soft tissue alterations were significantly different from 0 (*p* < 0.001). No statistically significant difference in soft tissue alterations was found between the 3-month and 6-month time points.

**Table 3:** The correlation between gingival thickness and soft tissue alterations.

Time point		GT_2_		GT_3_	
		*r*	*p* values	*r*	*p* values
3 months (T_1_)	MR	0.119	0.532	0.284	0.128
	HCC_2_	−0.084	0.660	−0.099	0.602
	HCC_3_	−0.165	0.384	−0.170	0.369
	MPR	−0.243	0.195	−0.251	0.181
	DPR	0.263	0.160	0.086	0.650
6 months (T_2_)	MR	0.066	0.729	0.276	0.140
	HCC_2_	−0.133	0.484	−0.161	0.394
	HCC_3_	−0.167	0.379	−0.136	0.473
	MPR	−0.281	0.133	−0.283	0.130
	DPR	−0.183	0.333	−0.241	0.200

r, Spearman’s rank correlation; GT_2_, the gingival thickness measured at 2 mm apical to the gingival zenith at baseline; GT_3_, the gingival thickness measured at 3 mm apical to the gingival zenith at baseline; HCC_2_, the horizontal contour collapse measured at 2 mm apical to the gingival zenith at T_0_; HCC_3_, the horizontal contour collapse measured at 3 mm apical to the gingival zenith at T_0_; MPR, mesial papilla recession; DPR, distal papillary recession.

Analyses presented in Table 3 are exploratory in nature and are not adjusted for potential data clustering.

## 4 Discussion

The results of the present study demonstrated that the soft tissue alterations following IIPP were similar for different gingival phenotypes, no statistically significant association was detected between gingival thickness and soft tissue changes following IIPP over a 6-month period.

IIPP has become a common procedure in clinical practice and exhibited a high survival rate. The present study reported a short-term survival rate of 100%, which is consonant with previous studies. A 1-year follow-up prospective study showed a survival rate of 100% with IIPP (26). Kan et al. achieved a cumulative implant success rate of 100% with IIPP during a follow-up ranging from 2 to 8 years (17). A long-term follow-up study reported that IIPP could present a 10-year survival rate of 90.9% (12). Besides the survival rate, soft tissue performance has become an important part to evaluate the clinical outcomes of IIPP (10). Thus, various methods have been proposed to assess soft tissue changes. Midfacial soft tissue level and papilla level could be measured intraorally using a periodontal probe (27). De Rouck et al. improved the accuracy of intraoral measurements by using a standardized stent as landmarks (28). Besides, clinical pictures could be used for measurements, and this method showed high reliability (29). Recently, the application of digital technology has provided us with a more accurate method for measuring soft tissue alterations. Superimposing the digital surface models at different time points enabled us to measure not only the linear changes in soft tissue level but also the profilometric soft tissue changes (26, 30–32). As optical scanning is very accurate and reliable, superimposing digital surface scans was considered the most accurate method for assessing soft tissue alterations (21, 33). However, few studies assessed the soft tissue alterations of different gingival phenotypes following IIPP through superimposing the digital surface models, and there was a lack of information about the profilometric soft tissue changes in different gingival phenotypes. Thus, superimposing the digital surface models was used in the present study to observe the soft tissue dynamic alterations following IIPP in different gingival phenotypes, and this study also confirmed the high reliability of this method, as the ICC values for the measurements were greater than 0.95 in the study.

The midfacial recession was one of the most common complications with IIPP. The present study found an overall midfacial recession of 0.81 ± 0.60 mm at 6 months. It is similar to the previous studies. Jiang et al. found that the midfacial recession following IIPP at 6 months was 0.63 ± 0.55 mm in a randomized clinical trial (34). De Rouck et al. also observed a midfacial recession of 0.54 ± 0.77 mm at 6 months (28). A one-year follow-up prospective clinical study showed a midfacial recession of 0.55 ± 0.53 mm following IIPP (35). For the horizontal contour collapse, the present study showed a significant tissue collapse during the healing period, while the HCC_2_ was 1.10 ± 0.60 mm and the HCC_3_ was 1.01 ± 0.53 mm at 6 months. Our results are also in line with those of Jiang et al., and they reported a mean collapse of approximately 1 mm at level 2–5 mm apical to the gingival margin during a healing period of 6 months (34). Besides, papilla recession was also found in the study (at 6 months: MPR: 1.03 ± 0.55 mm, DPR: 0.87 ± 0.38 mm), which is close to the previous studies (19, 35). In terms of the trend of soft tissue alteration, the midfacial recession, papilla recession, and horizontal contour collapse were mainly observed in the first 3 months and no significant changes were detected between the 3 and 6 months in the present study. The trend of soft tissue alteration was also concurrent with the previous studies (26, 28, 36). A systematic review evaluated the soft tissue alterations at 3, 6, and 12 months following IIPP and pointed out that the soft tissue alterations mainly took place in the first 3 months and then tended to stabilize (6).

One major controversial point was whether the gingival phenotype or gingival thickness exerts an impact on soft tissue alterations following IIPP. Different classifications of gingival biotype or gingival phenotype were used in the previous studies. For example, Ross et al. classified gingival thickness of < 1.5 mm as thin gingival phenotype (36), while Kan et al. categorized the gingival phenotype through the visibility of the periodontal probe (17). The between-study heterogeneity in the classification of gingival phenotypes might lead to differences in reported outcomes. According to a recent consensus of the 2017 World Workshop on the Classification of Periodontal and Peri-Implant Diseases and Conditions, the visibility of a periodontal probe which was inserted in the sulcus was recommended to evaluate the gingival phenotype: visible: thin (gingival thickness ≤1 mm); invisible: thick (gingival thickness > 1 mm). This classification criterion was used in the present study, and the gingival thickness measured at 2 mm below the gingival margin in the thin phenotype group or thick phenotype group was also in accordance with the consensus. This further indicates that a 2 mm sub-gingival measurement is a reliable indicator for gingival phenotyping. In the present study, no statistically significant difference in midfacial recession, papilla recession, and horizontal contour collapse were detected between thin and thick phenotypes. Beyond this, the correlation between gingival thickness and soft tissue alterations was also analyzed, and no statistically significant association was found in the current study. Differed from our study, Kan et al. reported that the thin phenotype presented greater midfacial recession at 1 year following IIPP (17). However, Evans et al. documented that the thin phenotype did not exhibit a significantly greater mid-facial recession as compared to the thick phenotype during a 6- to 50-month follow-up (9). Concern gingival thickness instead of gingival phenotype, a prospective study that evaluated soft tissue changes following IIPP demonstrated that there was no significant association between midfacial recession and gingival thickness at 3, 6, and 12 months after surgery (19). Similar results to ours have been reported by a recent randomized clinical trial, this study assessed soft tissue alteration following IIPP with or without connective tissue graft and found that connective tissue graft was not able to minimize the midfacial recession during a 6-month follow-up (34). As the previous studies documented, the soft tissue alteration was also affected by other factors including the implant, socket anatomy, surgical protocol, and the use of provisional restorations (2, 10, 13–16). A 5-year retrospective study demonstrated that gingival phenotype was not a significant factor when using a narrow-diameter implant with a correct three-dimensional implant position (36).

In this study, we strictly controlled the indications and followed standardized clinical protocols. Specifically, digital surgical guides were used to ensure precise implant placement and an adequate jumping gap, deproteinized bovine bone mineral was grafted into the jumping gap, and provisional restorations were utilized to provide soft tissue support. These strict indications and standardized procedures may have mitigated the impact of gingival thickness on soft tissue changes following IIPP, thereby resulting in similar soft tissue outcomes between the thin and thick gingival biotype groups. This also suggests that, in routine clinical practice, predictable clinical outcomes can be achieved for patients with a thin gingival phenotype undergoing IIPP, provided that strict indications are met and standardized protocols are followed.

Regarding the research methods, certain limitations need to be addressed. The first limitation is the lack of three-dimensional information about soft tissue alterations. However, considering the acceptance of clinicians, two-dimensional assessments were performed in the present study as they can provide the most intuitive and clear information for clinical practice. To ensure the reliability of the measurements, the assessor underwent standardization and training prior to the commencement of the study. All intraclass correlation coefficient (ICC) values for the measurements were equal to or exceeded 0.95, demonstrating high reliability and repeatability. Assessor blinding was implemented to mitigate the risk of bias. In addition, as this study focused exclusively on IIPP without a delayed implant placement control group, our conclusions regarding outcomes across different gingival phenotypes lack a direct comparative context. Furthermore, it should be noted that this study employed strict inclusion and exclusion criteria, with all patients undergoing a consistent treatment protocol performed by the same experienced clinician. Specifically, with the aid of digital technology, all implants were placed at a depth of 4–5 mm apical to the gingival margin, and the buccal gap dimension was maintained at >1 mm. While these standardized protocols and strict criteria minimized the confounding effects of unmeasured site-specific variables (e.g., buccal bone phenotype, implant depth, and buccal gap dimensions), they inevitably limit the generalizability of our findings. Therefore, future studies should explicitly account for these variables, and the results of this study should be interpreted with caution when applied to actual clinical settings. Notably, the sample size was determined assuming independent samples, but the analysis employed a mixed model, which could potentially compromise the statistical power. Another possible weakness of the present study is the short follow-up period of 6 months and the small sample size, as the study was powered to detect a 1 mm difference, it was underpowered to confirm or exclude the observed 0.16 mm difference. Future long-term prospective studies are needed to confirm the impact of gingival thickness on soft tissue alterations.

Within the constraints of this study, no statistically significant differences in soft tissue alterations were observed between different gingival phenotype groups at 6 months following IIPP. Since the study was powered to detect a clinically relevant difference of 1 mm, it lacked the sensitivity to definitively confirm or rule out the more subtle difference observed.

## Data Availability

The original contributions presented in the study are included in the article/Supplementary material, further inquiries can be directed to the corresponding author.
