# Supplementary material for: Influence of gingival thickness on soft tissue changes after immediate implants and immediate provisionalization in the esthetic zone: an ambispective cohort study
Source: Front Med (Lausanne). 2026 Aug 19;13:1888629. doi: 10.3389/fmed.2026.1888629 (PMC13533723; doi:10.3389/fmed.2026.1888629)
Supplement: Supplementary file 1 [file Table_1.docx]

**Table S1.** Results of overall soft tissue alterations at each time point (Mean ± SD, mm)

| **Parameter** | **3 months (T_1_)** | **6 months (T_2_)** | ***P* Values** |
| --- | --- | --- | --- |
| **MR** | 0.74 ± 0.65 | 0.81 ± 0.60 | 0.615 |
| **HCC_2_** | 1.10 ± 0.57 | 1.10 ± 0.60 | 0.997 |
| **HCC_3_** | 0.97 ± 0.49 | 1.01 ± 0.53 | 0.642 |
| **MPR** | 0.98 ± 0.43 | 1.03 ± 0.55 | 0.566 |
| **DPR** | 0.80 ± 0.43 | 0.87 ± 0.38 | 0.233 |

Abbreviations: SD, standard deviation; MR, midfacial recession; HCC_2_, the horizontal contour collapse measured at 2 mm apical to the gingival zenith at T_0_; HCC_3_, the horizontal contour collapse measured at 3 mm apical to the gingival zenith at T_0_; MRP, mesial papilla recession; DPR, distal papillary recession.
